# The Effects of Sex, Oral Contraception, and Menstrual Cycle Phase on Intraocular Pressure, Central Corneal Thickness, and Foveal Thickness: A Descriptive Analysis

**DOI:** 10.3390/vision5040048

**Published:** 2021-10-18

**Authors:** Lourdes Fortepiani, Brian K. Foutch, Molly R. Wilson

**Affiliations:** 1 Rosenberg School of Optometry, University of the Incarnate Word, San Antonio, TX 78229, USA; fortepia@uiwtx.edu (L.F.); MRWilson2020@yahoo.com (M.R.W.); 2 Department of Cellular and Integrative Physiology, UT Health San Antonio, San Antonio, TX 78229, USA; 3 Omni Vision, San Antonio, TX 78245, USA

**Keywords:** sex differences, oral contraception, menstrual cycle, intraocular pressure, corneal thickness, foveal thickness

## Abstract

The primary goal of this study was to investigate the effects of sex, oral contraceptive (OC) use, and menstrual cycle phase on common ocular parameters assessed during ophthalmic evaluations, namely intraocular pressure (IOP), central corneal thickness (CCT), and foveal thickness (FT), in young healthy adults. We measured IOP, CCT, and FT in 60 participants (16 men, 16 contraceptive users, and 28 cycling women) over two sessions that characterized the menstrual cycle phase in women. For men in our study, two sessions were separated by two weeks. For women, the two sessions were scheduled during the follicular and luteal phases of the menstrual cycle. There was a trend towards higher IOP in men, and the difference was significant for white, non-Hispanic subjects and for white subjects considered separately. There was also a trend for thicker corneas in women, but men had significantly thicker foveae. CCT and FT were not different between men and OC-users, hinting at a moderating hormonal effect of oral contraceptive use. We found that IOP, CCT, and FT were equivalent between the follicular and luteal phases, which may be owing to the timing of our sessions. However, our findings strongly suggest that clinicians should consider contraceptive use during routine ophthalmic evaluations, as it could inform glaucomatous risk in women.

## 1. Introduction

Normal intraocular pressure (IOP) is necessary for correct functioning of the human eye. Normal physiological values range from 10 to 21 mmHg, while daily IOP values vary by as much as 3 mmHg [1]. Despite this diurnal variation, IOP is typically highest in the morning, particularly right after waking [1,2]. Some factors known to affect IOP are age, gender, sex, stress, and obesity [3]. Sex hormones have also been proposed to impact IOP; however, while primary open angle glaucoma (POAG) is more prevalent in women than in men [4], there is equivocal evidence regarding sex differences in IOP values in non-glaucomatous individuals. Studies addressing the impact of sex hormones on IOP across the menstrual cycle or during hormone therapy in non-glaucomatous patients have also yielded unclear results [5,6,7].

Another factor that could contribute to variations in IOP is central corneal thickness (CCT). For example, there is a demonstrated positive correlation between CCT and IOP with increased prevalence of ocular hypertension in individuals with increased corneal thickness [8]. Corneal thickness may also change in response to hormonal influences such as menstrual cycle, pregnancy, menopause, and hormone therapy. There is at least one report of variations in CCT during the menstrual cycle [9]; corneas were thickest near the end of the cycle and thinnest at the beginning of the cycle. While the use of oral contraceptives seems to increase CCT [10], reports related to sex differences in corneal thickness are inconclusive.

In addition to the impact of CCT on IOP values, both significant increases in IOP and the prevalence of glaucoma have been associated with a decrease in foveal thickness (FT) [11]. Thinning of the macula has a direct impact on central vision and is considered an index of the structural progression of both primary open-angle and normotensive glaucoma [12]. Interestingly, recent studies have suggested a sexual dimorphism in macular thickness for IOP values within normal limits which was highly dependent on the demographics of the targeted population. Pakistani and black Afrikaner women exhibited thicker maculae than men [13,14], while the opposite findings were observed in Chinese and Indian subjects [15,16]; no difference between genders was reported in white Caucasians [17]. Macular structural changes may be under the influence of sex hormones, as the use of oral contraceptives has been linked to macular thinning [18].

Numerous attempts have been made to establish a clear correlation between sex and the ocular parameters involved in IOP regulation, but sex-related variability in IOP, CCT and FT and its relevant clinical impact remains unclear. The primary goal of this study was to investigate the effects of sex, oral contraceptive (OC) use, and menstrual cycle phase on IOP, CCT, and FT in young healthy men and women.

Previous investigations have uncovered thinner corneas in black subjects compared to white subjects [19,20], as well as higher IOP in Latinos of African ancestry [21]. Asefzadeh et al. also uncovered FT differences between white and black subjects [22], but they did not find racial differences in IOP. Others have shown sex differences in IOP in specific racial or ethnic groups. Large-scale studies revealed that Latino women had higher IOP than Latino men [23], Iranian men had higher IOP than Iranian women [24]. Considering this, we performed an exploratory analysis of IOP, CCT, and FT sex differences in subgroups self-identified by race (Asian, Black, South Asian, or White) and ethnicity (Hispanic or non-Hispanic).

Lastly, previous authors have also demonstrated associations between body mass index (BMI) and both IOP [25] and CCT [26,27]. Others have shown positive relationships between blood glucose levels (BGL) and IOP [28], CCT [27] and, more equivocally, FT [29]. A secondary goal of our study was then to explore our data for relationships between IOP, CCT, FT, and systemic parameters such as BMI, BGL, and systolic (SBP) and diastolic blood pressure (DBP).

## 2. Materials and Methods

### 2.1. Subjects

An a priori correlation power analysis using a small effect size (R^2^) of 0.16, power (1-β) = 0.8, and probability level (*p*) = 0.05 yielded a minimum sample size of 54 subjects, which became our recruitment goal. Exclusion criteria included a clinical history of diabetes, hypertension, thyroid disease, current oral or topical ophthalmic anti-inflammatory medication use, history of glaucoma, and refractive surgery. Due to its high prevalence among North American women [30], oral contraception was allowed for convenience in recruiting pre-menopausal women.

Sixty-eight individuals (51 women, 17 men) volunteered for the study. We excluded three women for chronic systemic anti-inflammatory use. Two volunteers (one man and one woman) had a history of diabetes and were also excluded. One female volunteer was excluded per a history of refractive surgery. Two women with unpredictable menstrual cycles were also excluded. All subjects were modestly compensated for their time and inconvenience after completing two sessions. Overall, 60 subjects (16 men, 44 women) completed the sessions. Twenty-eight of the women were experiencing monthly menstrual cycles with a mean length (±SD) of 29.7 ± 3.44 days. Sixteen women were using some oral form of hormonal contraception with monthly menstrual periods separated by 28.3 ± 1.10 days. The study protocol was approved by the institutional review board at the University of the Incarnate Word (UIW #14-05-002), and informed consent was obtained from 60 subjects. Subject age distributions are shown in Table 1. Median age was higher for men than women on independent samples median test (*p* = 0.001) but equivalent between OC users and cycling women (*p* = 0.528).

### 2.2. Sessions

Data collection sessions for men were scheduled two weeks apart. Women provided the start dates of their last three menstrual cycles to estimate the phase (follicular or luteal) of the reproductive cycle. Our goal was to collect data at two sessions. One session was scheduled during the first half of the cycle (i.e., follicular phase) and one during the second half cycle (around day 23, representing the midpoint of the luteal phase). For 16 of the 28 cycling women, we were informed at the start of their cycle and scheduled a session within the next week and a session approximately three weeks later. For the remaining 12 cycling women, sessions took place over two or three cycles. We scheduled oral contraceptive (OC) users in an identical manner. Twelve of the sixteen OC-users completed both sessions in the same month, and the remaining four completed the sessions over three months.

Data for the follicular phase were collected for all women between cycle days two and nine with an average day (mean ± S.D.) of 5.04 ± 1.81. Cycling women completed this session slightly later (day 5.50 ± 1.76) than OC-users (day 4.14 ± 1.61). Data for the luteal phase were collected between cycle days 17 and 26 with an average day (mean ± S.D.) of 22.3 ± 2.01, equivalent for cycling women (day 22.3 ± 1.93) and OC-users (day 22.4 ± 2.24). 

### 2.3. Procedures

As IOP values are diurnal in nature [2,31,32], we conducted all procedures between 7:00 and 9:00 a.m. To further dissuade participants with undiagnosed hypertension or diabetes, each session began with blood pressure (BP) and fasting blood glucose level (BGL) measurements. We also recorded the self-reported height and weight of each subject at their first session.

#### 2.3.1. Intraocular Pressure

The current gold standard for clinical IOP measurement is the Goldmann applanation tonometer (GAT), and the normal physiological values for IOP are based on this type of instrument [33]. However, rebound tonometers do not require the use of anesthetic and have been shown to correlate highly with GAT measures, even showing greater interrater reliability [34,35]. IOP was then measured using the Icare^®^ ic100 rebound tonometer (Icare USA, Raleigh, NC, USA), which averages six measurements to determine a mean measurement. Mean measurements were recorded for the right and left eyes at each session.

#### 2.3.2. Central Corneal Thickness

We determined CCT using a handheld (PachPen^®^) pachymeter (Accutome, Inc., Malvern, PA, USA). This model averages ten perpendicular corneal thickness measures to determine a mean central corneal thickness. Mean CCT was recorded for both the right and left eyes at each session.

#### 2.3.3. Foveal Thickness

We measured FT using a CirrusTM 4000 (Zeiss spectral domain optical coherence tomography (OCT) with a 512 × 128 (47 mm spacing) macular scan pattern. Scans with adequate reliability (i.e., signal strength ≥ 9/10) were saved for each eye by subject number and session, and the central subfield thickness was recorded as foveal thickness (FT) for each eye.

### 2.4. Data Analysis

We performed a multivariate analysis of variance (MANOVA; Pillai’s trace reported) with IOP, CCT and FT as outcome measures and with sex, OC use, and menstrual cycle phase (MCP; follicular vs. luteal) as fixed factors. We also investigated sex differences in IOP, CCT, and FT in all white subjects as well as in white non-Hispanics separately. We then investigated any differences in body mass index (BMI), fasting blood glucose levels (BGL), systolic blood pressure (SBP), and diastolic blood pressure (DBP) using the same fixed factors. Lastly, we performed exploratory correlations between outcome and screening measures. All analyses were performed using SPSS^®^ 25 (IBM^®^ Statistics, Armonk, NY, USA).

## 3. Results

### 3.1. Descriptive Results

All subjects completed at least one session. However, due to scheduling conflicts or unavailable clinical equipment, two women (one OC user, one cycling) were only able to complete one session. We were also missing IOP observations for one male subject and CCT and FT measures for two male subjects. While it has been recommended that investigators consider separate eyes as ‘within subjects’ factors in two-eye studies [36], it has long been recognized that it is not necessary to do so when the values are highly correlated [37]. Our inter-eye correlations were very high for IOP (r = 0.877, *p* < 0.001), CCT (r = 0.950, *p* < 0.001), and FT (r = 0.973, *p* < 0.001). Therefore, right and left eye values were averaged for IOP, CCT, and FT. See Table 2 for a summary of all mean values.

### 3.2. Intraocular Pressure, Central Corneal Thickness, and Foveal Thickness Analysis by Sex, Contraceptive Use and Menstrual Phase

**Figure 1:**
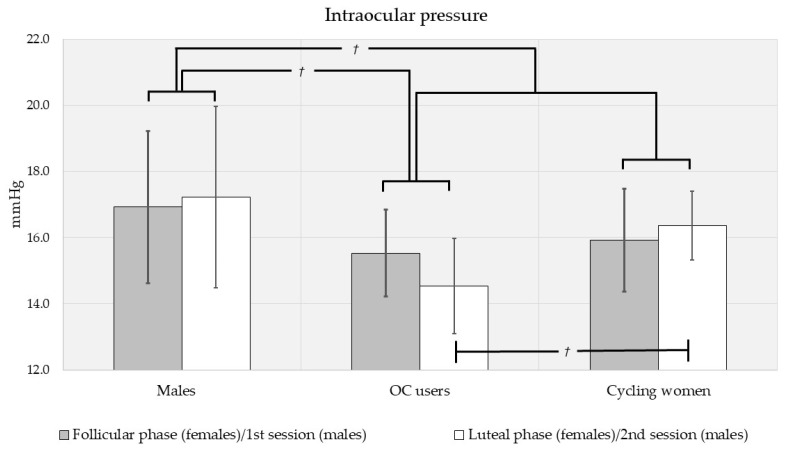
Intraocular pressures for men and women (shown separately by oral contraceptive use and menstrual cycle phase). There was a trend toward higher IOP in men and in cycling women during the luteal phase. On post hoc comparison, there was also a trend toward higher IOP in men than OC users. *†* Significant at the 0.10 level; Error bars represent 95% confidence intervals.

**Figure 2:**
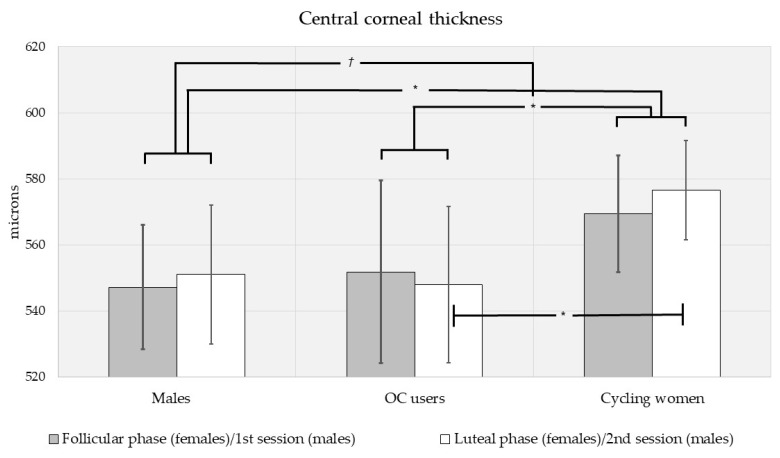
Central corneal thickness for men and women (shown separately by oral contraceptive use and menstrual cycle phase). There was a trend toward thicker central corneas in women. Corneal thickness was significantly higher in cycling women than OC users overall as well as during the luteal phase. On post hoc comparison, CCT was lower in men than cycling women. *†* Significant at the 0.10 level, * significant at the 0.05 level; Error bars represent 95% confidence intervals.

**Figure 3:**
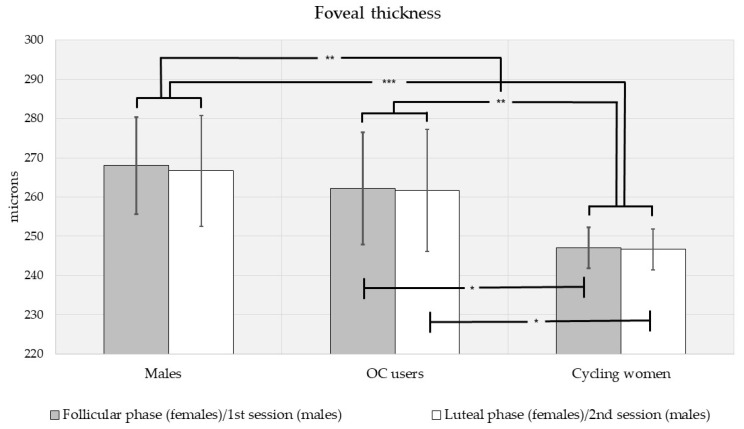
Foveal thickness for men and women (shown separately by oral contraceptive use and menstrual cycle phase). Men had thicker foveae than women, and OC users had higher thickness than cycling women overall as well as during the follicular and luteal phases. On post hoc comparison, FT was higher in men than cycling women. * Significant at the 0.05 level, ** significant at the 0.01 level, *** significant at the 0.001 level; Error bars represent 95% confidence intervals.

Biometric (IOP, CCT and FT) measures were different by sex, via multivariate analysis of variance (F[3,110] = 6.15, *p* < 0.001; see Figure 1, Figure 2 and Figure 3). Intraocular pressure was slightly higher in men than in women, but the difference did not reach significance (MD (mean difference) = 1.39 mmHg, *p* = 0.090; see Figure 1). There was a trend toward higher central corneal thickness in women (MD = 16.6 μm, *p* = 0.080; see Figure 2). Men had significantly higher FT (MD = 15.4 μm, *p* < 0.001; see Figure 3).

When compared by contraceptive use, biometric measures were different between oral contraceptive (OC) users and cycling women (F[3,78] = 5.17, *p* = 0.003). When compared by menstrual cycle phase, biometrics were essentially equivalent (F[3,78] = 0.050, *p* = 0.985; see Figure 1, Figure 2 and Figure 3). Similarly, pairwise comparisons revealed no differences in any measure (IOP, CCT, FT) by menstrual phase for OC users and cycling women. However, mean IOP was slightly lower in OC users than cycling women (MD = −1.36 mmHg, *p* = 0.179), and the trend was stronger during the luteal phase (MD = 1.67 mmHg, *p* = 0.076; see Figure 1). During the follicular phase, there was no effect of OC use on IOP. CCT was higher in cycling women than OC users (MD = 23.8 μm, *p* = 0.016). Considered separately by phase, CCT was higher in cycling women during the luteal phase (MD = 28.5 μm, *p* = 0.043) but not the follicular phase (MD = 17.6 μm, *p* = 0.282; see Figure 2). FT was higher in cycling women overall (MD = 15.3 μm, *p* = 0.002) as well as during the follicular (MD = 15.2 μm, *p* = 0.021) and luteal phases (MD = 15.1 μm, *p* = 0.035; see Figure 3).

We did not plan for comparisons between men and OC users or men and cycling women separately. However, trends in our data warranted such post hoc comparisons. When controlling for the multiple comparisons using Bonferroni post hoc adjustments, IOP tended to be lower in OC users than men (MD = −2.30 mmHg, *p* = 0.064), but IOP was equivalent between men and cycling women (MD = −0.946 mmHg, *p* = 0.845; see Figure 1). CCT was higher in cycling women than men (MD = 23.9 μm, *p* = 0.044) but equivalent for men and OC users (MD = 0.105 μm, *p* = 1.000; see Figure 2). FT was lower in cycling women than in men (MD = −20.6 μm, *p* < 0.001), but equivalent for men and OC users (MD = −5.33 μm, *p* = 1.000; see Figure 3).

### 3.3. Intraocular Pressure, Central Corneal Thickness, and Foveal Thickness Analysis by Sex, Race, and Ethnicity

Other than white subjects, there were too few subjects within racial groups to make useful comparisons by sex. There were also too few subjects in any subgroup to compare by contraceptive use or menstrual cycle phase. We were able to compare all white subjects as well as non-Hispanic white subjects by sex. Mean values (±SD) for all subgroups are shown in Table 3.

For all white subjects, IOP, CCT, and FT were different by sex via multivariate analysis of variance (F[3,88] = 3.93, *p* = 0.011). IOP was higher in men than women (MD = 2.02 mmHg, *p* = 0.033). CCT was equivalent between men and women (MD = −10.3 μm, *p* = 0.320), but there was a trend toward higher FT in men (MD = 8.72 μm, *p* = 0.097).

For white, non-Hispanic subjects, IOP, CCT, and FT measures were different by sex via multivariate analysis of variance (F[3,74] = 6.15, *p* < 0.001). IOP was higher in men than women (MD = 3.03 mmHg, *p* = 0.002). CCT was equivalent between men and women (MD = −6.62 μm, *p* = 0.570), but FT was higher in men (MD = 11.6 μm, *p* = 0.047).

### 3.4. Body Mass Index, Blood Glucose Level, and Blood Pressure Analysis by Sex, Contraceptive Use and Menstrual Phase

Consistent with previous large-scale findings [38], BMI values were not distributed normally in our subjects (Kolmogorov-Smirnov test, *p* < 0.001), but were distributed similarly between men, OC users, and cycling women (Mann–Whitney test, *p* = 0.085). Median BMI was higher for men (25.1 kg/m^2^) than women (22.2 kg/m^2^) on the independent samples median test (*p* = 0.025). BMI values were also higher for men than cycling women (22.5 kg/m^2^; *p* = 0.015) and OC users (21.5 kg/m^2^; *p* = 0.002) but equivalent between OC users and cycling women (*p* = 0.330).

Systemic parameters (BGL, SBP and DBP) were collected at the first session for all subjects but were measured at both sessions for some subjects. Mean values (±SD) are shown in Table 4. BGL, SBP and DBP measures were higher in men than women via multivariate analysis of variance (F[3,83] = 6.78, *p* < 0.001). BGL (MD = 10.5 mg/dL, *p* = 0.001), SBP (MD = 11.2 mmHg; *p* < 0.001), and DBP (MD = 4.98 mmHg; *p* = 0.020) were all higher in men than women.

When compared by contraceptive use, BGL, SBP, and DBP were equivalent between OC users and cycling women (F[3,63] = 1.65, *p* = 0.186). The only significant pairwise difference by OC use was that DBP was higher in OC users during the luteal phase (MD = 6.01 mmHg, *p* = 0.026). When compared by menstrual cycle phase, BGL, SBP, and DBP were equivalent (F[3,63] = 0.683, *p* = 0.566). Pairwise comparisons revealed no differences in BGL, SBP, or DBP between the follicular and luteal phases for either all women, OC users, or cycling women.

### 3.5. Relationships between Ocular Biometrics and Systemic Parameters

Lastly, we examined the relationships between biometric variables (IOP, CCT, and FT) as well as between the biometrics and systemic parameters (BMI, BGL, SBP, and DBP). The correlation values are summarized in Table 5. Relationships between systemic parameters were not investigated as they do not contribute to our central questions of sex, oral contraceptive use, or menstrual cycle phase differences in biometrics.

There was a positive association between IOP and CCT for all subjects (r = 0.401, *p* < 0.001) as well as for men (r = 0.446, *p* = 0.014), all women (r = 0.457, *p* < 0.001), and cycling women (r = 0.621, *p* < 0.001). There was no association between IOP and CCT for OC users (r = 0.116, *p* = 0.549). IOP was not significantly associated with any other ocular biometric or systemic parameters.

CCT was negatively correlated with FT for all subjects (r = −0.224, *p* = 0.015), but the relationship only held for men (r = −0.473, *p* = 0.008). CCT was also negatively associated with BGL for all subjects (r = −0.262, *p* = 0.012). While CCT and BGL were negatively associated for all groups, the relationship was only significant for cycling women (r = −0.301, *p* = 0.050). While there were relationship trends between CCT and SBP for all subjects (r = −0.175, *p* = 0.094), the only statistically significant finding was for cycling women (r = −0.379, *p* = 0.010).

FT was positively correlated with BGL (r = 0.360, *p* < 0.001), SBP (r = 0.363, *p* < 0.001), and DBP (r = 0.346, *p* < 0.001) for all subjects. Similar relationships with FT held for men for BGL (r = 0.779, *p* < 0.001), SBP (r = 0.635, *p* = 0.005), and DBP (r = 0.628, *p* = 0.005). We found no such associations between FT and systemic parameters in women overall. There were positive correlations between FT and both BP measures in cycling women, but they did not reach statistical significance. Similarly, FT was positively associated with BGL (r = 0.344), but the relationship was not statistically significant (*p* = 0.063).

## 4. Discussion

Elevated intraocular pressure (IOP) is an important risk factor for developing glaucoma [39]. IOP is routinely measured clinically as it can affect the classification and management of glaucoma [40], but sex-related variations in ocular biometrics are not usually considered during ophthalmic exams. The use of oral supplements or therapies in the female population is part of the information collected during history taking, but menstrual cycle phase on IOP readings is not currently factored into clinical decisions. While we observed no clear sexual dimorphism in IOP or CCT, trends in our results suggest that sex may be useful to consider, especially when combined with contraceptive use. We only found sex differences in CCT when oral contraceptive use was considered, as CCT was lower in men than in cycling women. Our most robust findings were for FT, which was significantly higher in men than in women. These relationships for CCT and FT only held when comparing men and cycling women, as CCT and FT were equivalent between men and OC users. These findings further implicate the need to consider contraceptive use in CCT and FT differences.

We did find significantly higher IOP in men than women when considering white subjects and white non-Hispanic subjects separately. Similar reports from populations from different geographical areas have yielded inconsistent results. In contrast to our findings in white subjects, IOP was slightly higher in women than in men in studies performed in Middle Eastern, black, and Latino populations using either Goldman applanation or non-contact tonometers [23,24,41]. We also found studies in Nigerian and German populations that failed to report a clear sex difference in IOP [42,43]. Results from studies in Italian, Spanish, and Korean populations compare with our findings, with higher IOP in men, mostly using applanation tonometry [44,45,46]. We used a rebound tonometer, so IOP measurement method could be a factor in comparing our results to these equivocal findings. However, we can find groups with similar findings that use different tonometer types; thus, it is unlikely that this difference can be based only on methodology. It appears likely that race and ethnicity should be considered in future investigations of sex dimorphism in IOP.

It is not unusual to consider the phase of the menstrual cycle to assess hormonal variations. IOP and CCT values have long been known to fluctuate during the menstrual cycle [5], but reliable correlations between IOP or CCT and the different phases of the cycle or the levels of sex hormones have not been clearly established. This is despite numerous attempts over the years [5,6]. In our population, we also found minimal variations in IOP, CCT, or FT between follicular and luteal phases.

Hormone therapy is widely used with different therapeutic goals, including contraception [30,47]. When OC use was considered as a factor in our study, IOP was slightly lower in OC users than in cycling women, with a stronger trend during the luteal phase (Figure 1). Our results contradict reports that the use of hormone contraceptives is associated with increased IOP [7]. In a prospective analysis in the Nurses’ Health Study [48], five-year OC use was associated with a 25% increased risk of POAG; however, in our subjects OC had the opposite effect, lowering IOP. It should be noted that the Nurse’s Health Study addressed the impact of oral contraceptives on POAG but not IOP levels [48]. However, oral contraceptives have been associated with either lower IOP, as seen in our subjects [49], or no impact on IOP [50]. If we consider the frequent use of oral contraceptives [30], it seems pertinent to assess their use in the female population during clinical history recording. This is especially the case in patients at high risk for glaucoma.

It is known that CCT can affect the readings of IOP [51] and possibly mask the accurate diagnosis of glaucoma, but sex difference in corneal thickness and its effect on IOP readings are not currently considered clinically relevant. In our subjects, there was a trend toward higher CCT in women compared to men, but the finding did not reach statistical significance across all subjects. Perhaps our most telling finding for CCT is its positive relationship with IOP in young subjects. These results are similar to a previous study where clear sex differences were not found in IOP nor CCT, but CCT was still directly associated with IOP in young (20–34 yrs) subjects [52]. Galgauskas et al. (2010) only found this positive association in older (>50 yrs) subjects, and further found that the relationship increased in subsequent decades [53]. Combined with our findings, it seems that CCT is associated with IOP but not sex, and aging may strengthen the association. The impact of CCT on IOP has been shown to be even more evident in individuals with ocular hypertension [8], implicating the importance of CCT when measuring IOP in all patients.

The lack of significant CCT sex differences across all subjects does not rule out sexual dimorphism in the relationship of CCT with IOP or FT. CCT was negatively correlated with FT across all our subjects. The relationship was even stronger in men, but not found in women. We also found positive correlations between IOP and CCT for all subject groups except OC users. The latter finding could be due to a low number of OC users in our study, but the relationship held in the same number of male subjects. It could also be due to the variety of OC used by our subjects. It is as likely that the IOP/CCT relationship is simply disrupted in oral contraceptive use. Further, men had significantly thinner corneas than cycling women, further evidencing the need to consider contraceptive use in determining clinical glaucoma risk in women.

Combined with our findings, the impact of the menstrual cycle on variations in CCT also seems controversial. Some studies have reported variations [9], but others have failed to show CCT differences during any two points in the menstrual cycle [54]. Furthermore, it seems that oral contraceptives increase CCT [10], which could lead to misdiagnosis of glaucoma. Contrary to these outcomes, we found that corneal thickness was thinner in OC users than in cycling women. Whether this difference in findings was due to OC composition or the time when the measurements were taken is unclear. Unfortunately, there is a scarcity of studies evaluating the impact of oral contraceptive use on CCT. The duration of oral contraceptive use is an important factor for predicting glaucoma, with an increased risk after 3–5 years of use [48]; however, it is unknown if this impact is mediated by altered CCT. Different glaucoma medications have a different impact on CCT despite their lowering IOP effect. Prostaglandin analogs decrease CCT [55], while topical beta blockers increase CCT [56]. A longitudinal study might be necessary to correlate oral contraceptive use with ocular biometrics (such as CCT) and IOP.

Our most robust sex differences were found in FT. In our study, men had thicker foveae than women and cycling women. Recent studies have suggested a sexual dimorphism in macular thickness for IOP values within normal limits, but the direction of the difference is highly dependent on the specific population. Our findings compared with those observed in Chinese and Indian women [15,16], but not with other investigators who observed thicker FT in Pakistani [13] and black Afrikaner [14] women. At least one study has shown that Hispanic women had thinner foveae than men [57]. However, we were unable to demonstrate differences in FT between Hispanic men and women. Because of the small sample size, we only attempted to differentiate between men and women in white subjects and in white, non-Hispanic subjects. We found that men had higher foveal thickness than women in both populations. The findings in white subjects contrast with those of Chan et al., who found no FT sex differences in Caucasians [17].

When the menstrual cycle phase was considered, we could not evidence FT changes between the follicular and luteal phases. There are similar reports of minimal variations in retinal nerve fiber layer (RNFL) or retinal thickness during the menstrual cycle using standard OCT [58] or OCT angiography (OCT-A) [59]. However, FT may be susceptible to exogenously administered sex hormones, as OC users in our study had thicker foveae than cycling women. Our results contrast with previous reported associations of macular thinning with OC use [18,60].

Overall, it seems that OC use moderated sex differences in CCT and FT in our study. While women had thicker corneas but thinner foveae than men for similar physiological IOP levels, the differences in CCT and FT were less in OC users than in cycling women. Thus, the use of oral contraceptives should be considered during routine ophthalmic evaluations as it could also inform glaucomatous risk in women. In addition, our results inform future investigations to consider IOP, CCT, and FT pre- and post-OC use.

Age has been shown to affect ocular biometrics, but OC users and cycling women were of a similar age in our study. Therefore, age was not likely a factor in our differences found by contraceptive use. However, men were older than women in our study, so age as well as the coincident anatomical changes could impact sex differences. We found significantly higher IOP in white men and white non-Hispanic men than in women, comparable to a previous study in a similar population [43]. However, a large-scale study in a mainly white population showed that gender differences in IOP were no longer present when the data were age adjusted [61], hinting at an interaction between age and gender effects on IOP. Another study in a different demographic showed no age effects on IOP until after the age of 50 [62]. Since the age of our cohort was not over 50 years, we expect that age was not a likely contributor to our sex differences in IOP. Previous reports of age effects on CCT are also equivocal, with both age-related corneal thinning [27,63] and no age-related differences [64]. It is doubtful that our robust FT sex difference could be due to age differences. While there is evidence that average macular thickness thins at a rate of about 5% per decade (even in younger subjects like ours [65]), foveae were thinner in our younger female subjects.

All systemic parameters (BMI, BGL, SBP, and DBP) were significantly higher in men than in women, possibly mediating some of our sex differences in ocular biometrics. Our trends for higher IOP in men could have been due to higher BMI and BGL levels, which have both been found to positively correlate with IOP [25,28]. The role of BP in the regulation of IOP is complex, and some studies have shown that BP increases of 40 mmHg are only accompanied by IOP increases of 1 mmHg (reviewed by He et al. [66]). It is then difficult to imagine that small sex differences in SBP and DBP (<40 mmHg) could account for any sex differences in IOP in our study. In addition, while we found small positive associations between IOP and BMI, SBP, and DBP, no relationship approached significance. However, FT in our subjects was positively associated with BGL, SBP, and DBP in all subjects and in men. Few studies have evaluated systemic effects on foveal thickness, but at least one has shown an association between systemic hypertension and changes in macular thickness [67]. These results support the need to capture systemic parameters in studies of ocular biometrics.

### Limitations

Our comparisons were limited by a relatively low number of subjects. In addition, the distribution was unequal with more cycling women than men and contraceptive users. However, the relatively low participation rate was not expected in this type of study. Data collection times were inconvenient, particularly as sessions were held in the early morning and sampled mainly from a student population and their friends and family. There was also no academic incentive to participate, further limiting student involvement.

There are limitations regarding oral contraceptive (OC) users in our study. First, we failed to collect information on when participants started oral contraceptives. This obviously limits any inferences that OC use reorganizes any of our measured outcomes, as we did not have a reference IOP value from our participants prior to OC use. Second, the composition of OC has changed over the years [47], complicating the establishment of a clear correlation between hormone levels and our outcomes (IOP, CCT, and FT). Additionally, exogenous hormone levels in current OC types do not correlate with physiological hormone levels during the menstrual cycle [68]. We further learned during initial recruitment that subjects were using mono-, bi-, or tri-phasic oral contraceptives. All types deliver a synthetic form of estrogen (ethinyl estradiol) and one of several types of synthetic progesterone (i.e., progestins) that differ both in hormone dosage and central nervous system effects [68,69]. The use of any OC compromises the ovarian hypothalamic axis and inhibits the pulsatile production and release of endogenous sex hormones, and most utilize a placebo week to allow for menses [70]. Thus, it is unclear whether the trends observed in IOP (or the significant effects on CCT and FT) are due to OC composition rather than its impact on endogenous hormone levels. Lastly, as hormone levels typically peak approximately two hours after ingesting oral contraceptives [71], it would have been important to capture the time the pill was taken. However, these considerations do not affect our differences found in comparing men with cycling women, nor do they limit inferences that OC users in this study, as a group, have thinner central corneas but thicker foveae.

Mostly absent in our findings are differences between follicular and luteal phase outcomes. The follicular phase is from days 1–14, and the luteal (or secretory) phase is from days 15–28. The follicular phase contains both the menstrual (days 1–5) and proliferative (days 6–14) phases. While our IOP findings are consistent with at least one study [6], Adhikari et al. [72] found IOP to be lowest during the follicular phase. However, Adhikari considered three phases in cycling women (menstrual, proliferative, and secretory), restricting IOP measurements of the menstrual phase to days 1–5, early in the follicular phase. Our data collection for cycling women took place later in the follicular phase, on average at day 5. It is possible that hormone levels, particularly estradiol, increased sufficiently to moderate any IOP differences between the follicular and luteal phases. After all, IOP differences were like those found by Adhikari between the proliferative and secretory phases [72]. The lack of a cyclical effect on CCT also contrasts with previous findings of cyclical CCT differences [9,73]. CCT in both of those studies was also collected on different days than in our study, i.e., very early (<day 3) and very late (>day 27) in the cycle. Future investigations using two menstrual cycle phases should then be careful to collect data early in the follicular phase and late during the luteal phases of the menstrual cycle.

## 5. Conclusions

Our investigation into the effects of sex, oral contraceptive use, and menstrual cycle phase did not establish a clear IOP difference between men and women. Despite a small number of subjects, our data revealed a trend toward higher IOP in men. This IOP sex difference was significant when white subjects and white non-Hispanic subjects were considered separately, informing clinicians to consider other demographics in sex differences in IOP. Oral contraceptive use appeared to moderate sex differences in corneal and foveal thickness, as cycling women had significantly thicker corneas but thinner foveae than both men and OC users. Lastly, we found no differences in IOP, corneal thickness, or foveal thickness between the follicular and luteal phases. Further studies should be careful to collect data early in the menstrual cycle to better isolate the hormonal differences between the follicular and luteal phases.

## Figures and Tables

**Table 1:** Age statistics for all study participants.

	Range	Mean ± S.D.	Median
All subjects (*n* = 60)	20–50 yrs	25.9 ± 5.06 yrs	24.0 yrs
Men (*n* = 16)	22–50 yrs	27.9 ± 6.44 yrs	26.5 yrs
Women (*n* = 44)	20–44 yrs	25.2 ± 4.22 yrs	24.0 yrs
- Cycling (*n* = 28)	21–44 yrs	25.8 ± 5.01 yrs	24.0 yrs
- Oral contraceptive users (*n* = 16)	20–28 yrs	23.9 ± 1.72 yrs	24.0 yrs

**Table 2:** Mean values (±SD) for all ocular parameters.

	IOP	CCT	FT
	(mmHg)	(μm)	(μm)
All subjects, *n* = 60	16.0 ± 3.90 (117)	560 ± 44.7 (116)	256 ± 23.3 (116)
Men, *n* = 16	17.1 ± 4.97 (31)	549 ± 38.6 (30)	267 ± 25.6 (30)
- 1st session	16.9 ± 4.70 (16)	547 ± 38.5 (16)	268 ± 25.2 (16)
- 2nd session	17.2 ± 5.40 (15)	551 ± 40.1 (14)	267 ± 26.9 (14)
Women, *n* = 44	15.7 ± 3.38 (86)	564 ± 46.1 (86)	252 ± 21.2 (86)
- Follicular	15.6 ± 3.79 (43)	564 ± 49.4 (43)	252 ± 20.5 (43)
- Luteal	15.7 ± 2.97 (43)	566 ± 43.7 (43)	252 ± 22.1 (43)
Cycling women, *n* = 28	16.1 ± 3.54 (55)	573 ± 43.6 (55)	247 ± 13.9 (55)
- Follicular	15.9 ± 4.19 (28)	569 ± 47.5 (28)	247 ± 14.1 (28)
- Luteal	16.4 ± 2.76 (27)	577 ± 39.8 (27)	247 ± 13.9 (27)
OC-users, *n* = 16	14.8 ± 2.95 (31)	549 ± 47.2 (31)	262 ± 27.8 (31)
- Follicular	15.1 ± 2.95 (15)	551 ± 50.8 (15)	262 ± 26.5 (15)
- Luteal	14.5 ± 3.02 (16)	547 ± 45.1 (16)	261 ± 29.8 (16)

( ) = number of measurements, OC = oral contraceptive, IOP = intraocular pressure, CCT = central corneal thickness, FT = foveal thickness.

**Table 3:** Mean values (±SD) for all ocular parameters by race, ethnicity, and sex.

	IOP	CCT	FT
	(mmHg)	(mm)	(mm)
Asian, *n* = 5	14.6 ± 2.45 (10)	556 ± 49.1 (10)	265 ± 31.4 (10)
- Men, *n* = 3	14.6 ± 2.89 (6)	541 ± 59.3 (6)	279 ± 33.7 (6)
- Women, *n* = 2	14.5 ± 2.04 (4)	579 ± 14.6 (4)	243 ± 6.56 (4)
Black, *n* = 2 (all women)	17.5 ± 3.76 (4)	608 ± 52.1 (4)	239 ± 15.6 (4)
South Asian, *n* = 5 (all women)	15.1 ± 4.12 (10)	562 ± 47.3 (10)	238 ± 16.2 (10)
White, *n* = 48	16.2 ± 4.00 (93)	559 ± 43.2 (92)	258 ± 22.1 (92)
- Men, *n* = 13	17.7 ± 5.22 (25)	551 ± 33.1 (24)	265 ± 23.1 (24)
Hispanic, *n* = 2	12.0 ± 5.60 (4)	525 ± 19.0 (4)	241 ± 12.0 (4)
Non-Hispanic, *n* = 11	18.8 ± 4.51 (21)	556 ± 33.1 (20)	269 ±21.9 (20)
- Women, *n* = 35	15.7 ± 3.33 (68)	561 ± 46.2 (68)	256 ± 21.5 (68)
Hispanic, *n* = 5	14.9 ± 3.07 (10)	554 ± 54.8 (10)	245 ± 11.2 (10)
Non-Hispanic, *n* = 30	15.8 ± 3.38 (58)	562 ± 45.0 (58)	258 ± 22.3 (58)

( ) = number of measurements, IOP = intraocular pressure, CCT = central corneal thickness, FT = foveal thickness.

**Table 4:** Summary of all systemic parameters.

	* BMI	BGL	SBP	DBP
	(kg/m^2^)	(mg/dL)	(mmHg)	(mmHg)
All subjects	22.5; 6.50 (59)	85.8 ± 12.2 (89)	117 ± 12.0 (91)	72.7 ± 8.09 (90)
Men	25.1; 6.90 (16)	94.4 ± 12.8 (18)	126 ± 17.1 (18)	76.8 ± 9.10 (18)
- 1st session	--	90.4 ± 11.3 (9)	125 ± 15.9 (9)	74.7 ± 9.55 (9)
- 2nd session	--	98.4 ± 13.6 (9)	127 ± 19.1 (9)	78.9 ± 8.64 (9)
Women	22.2; 4.10 (43)	83.7 ± 11.1 (71)	114 ± 9.22 (73)	71.7 ± 7.57 (72)
- Follicular	--	85.2 ± 11.1 (36)	114 ± 10.2 (37)	71.7 ± 7.45 (36)
- Luteal	--	82.8 ± 10.4 (35)	115 ± 8.51 (36)	71.5 ± 7.90 (36)
Cycling women	22.5; 4.40 (28)	85.7 ± 11.1 (43)	114 ± 9.50 (45)	70.4 ± 6.44 (44)
- Follicular	--	86.9 ± 11.4 (22)	115 ± 10.6 (23)	71.7 ± 10.6 (22)
- Luteal	--	84.4 ± 10.8 (21)	113 ± 8.41 (22)	69.1 ± 6.14 (22)
OC users	21.5; 7.30 (15)	80.7 ± 10.7 (28)	116 ± 8.85 (28)	73.7 ± 8.72 (28)
- Follicular	--	82.6 ± 10.4 (14)	114 ± 9.95 (14)	71.7 ± 8.89 (14)
- Luteal	--	80.4 ± 9.77 (14)	118 ± 8.08 (14)	75.4 ± 9.02 (14)

* Median values; interquartile range, ( ) = number of measurements, OC = oral contraceptive, BMI = body mass index, BGL = fasting blood glucose level, SBP = systolic blood pressure, DBP = diastolic blood pressure. -- = no data.

**Table 5:** Correlations between ocular biometrics and systemic parameters (Pearson’s r reported).

	All Subjects	Men	Women	Cycling Women	OC Users
IOP vs.	--	--	--	--	--
- CCT	0.401 (<0.001)	0.446 (0.014)	0.457 (<0.001)	0.621 (<0.001)	0.116 (0.549)
- FT	−0.065 (0.482)	−0.214 (0.255)	−0.061 (0.750)	−0.101 (0.461)	0.078 (0.688)
- BMI *^†^*	0.153 (0.327)	0.279 (0.314)	0.059 (0.707)	0.099 (0.616)	0.166 (0.554)
- BGL	−0.046 (0.665)	0.069 (0.786)	−0.048 (0.686)	−0.077 (0.625)	−0.161 (0.396)
- SBP	0.057 (0.589)	0.224 (0.371)	0.029 (0.803)	0.066 (0.667)	0.027 (0.886)
- DBP	0.036 (0.735)	0.295 (0.234)	−0.023 (0.843)	0.113 (0.464)	−0.083 (0.663)
CCT vs.	--	--	--	--	--
- FT	−0.224 (0.015)	−0.473 (0.008)	0.078 (0.481)	−0.105 (0.446)	0.011 (0.955)
- BMI *^†^*	−0.113 (0.394)	0.001 (0.998)	−0.062 (0.693)	0.017 (0.932)	−0.254 (0.402)
- BGL	−0.262 (0.012)	−0.397 (0.103)	−0.162 (0.172)	−0.301 (0.050)	−0.175 (0.355)
- SBP	−0.175 (0.094)	0.034 (0.895)	−0.176 (0.131)	−0.379 (0.010)	0.098 (0.607)
- DBP	−0.046 (0.666)	−0.121 (0.631)	0.038 (0.746)	−0.184 (0.231)	0.337 (0.068)
FT vs.	--	--	--	--	--
- BMI *^†^*	0.113 (0.394)	0.235 (0.381)	−0.020 (0.899)	0.010 (0.960)	0.102 (0.718)
- BGL	0.360 (<0.001)	0.779 (<0.001)	0.125 (0.292)	0.019 (0.902)	0.344 (0.063)
- SBP	0.363 (<0.001)	0.635 (0.005)	0.102 (0.384)	0.250 (0.097)	−0.023 (0.904)
- DBP	0.346 (<0.001)	0.628 (0.005)	0.176 (0.133)	0.276 (0.070)	0.057 (0.765)

*^†^* For BMI, rank correlations with Spearman’s ρ shown. ( ) = *p*-value, IOP = intraocular pressure, CCT = central corneal thickness, FT = foveal thickness, OC = oral contraceptive, BMI = body mass index, BGL = fasting blood glucose level, SBP = systolic blood pressure, DBP = diastolic blood pressure. -- = no data.

## Data Availability

Portions of the data presented in this study may be made available on request from the corresponding author. The data are not publicly available due to health privacy restrictions.
